# Soil organic carbon and nitrogen storage under a wheat (*Triticum aestivum* L.)—maize (*Zea mays* L.) cropping system in northern China was modified by nitrogen application rates

**DOI:** 10.7717/peerj.13568

**Published:** 2022-06-30

**Authors:** Lifang Wang, Shijie Liu, Geng Ma, Chenyang Wang, Jutao Sun

**Affiliations:** 1 State Key Laboratory of Wheat and Maize Crop Science, Henan Agricultural University, Zhengzhou, China; 2 Henan Technology Innovation Center of Wheat, Henan Agricultural University, Zhengzhou, China; 3 College of Tobacco Science, Henan Agricultural University, Zhengzhou, China

**Keywords:** Nitrogen application, Soil carbon and nitrogen storage, Wheat-maize cropping, Yield, North China Plain

## Abstract

Field cultivation practices have changing the carbon and nitrogen cycles in farmland ecosystem, soil organic carbon (SOC) and total nitrogen (TN) were the important parameters in maintaining soil quality and increasing agricultural productivity, however, N application’s effects on the SOC and TN storage capacity under intensive wheat-maize cropping system remain unclear. Therefore, we investigated the characteristics and relationships of SOC and TN for wheat-maize cropping system under nitrogen treatments. In doing so, continuous applications of four nitrogen application rates were examined: 0, 180, 240 and 300 kg ha^−1^ (N0, N180, N240 and N300, respectively). Wheat yields under N180 and N240 were significantly higher than that under N300, while the maize yields under N180, N240 and N300 were significantly higher than that under N0 by 79.79, 85.23 and 86.85%, respectively; the TN content and storage were significantly higher under N240 than that under other N levels in 40–60 cm soil layer after wheat growing season; the SOC content and storage under N180 and N240 were significant higher than that under N300 in 20–40 cm after maize growing season. The correlations between SOC and TN contents (or storage) were stronger after wheat planting than maize planting. These findings provide a basis for further studies on the effect of long-term N application on SOC and TN storage, crop quality and nitrogen use efficiency under wheat-maize cropping systems.

## Introduction

Soil organic carbon (SOC) is the most important component of the global carbon (C) cycle (Shah et al., 2021), while soil nitrogen (N) dynamics have considerable effects on the terrestrial carbon cycle. In this regard, conservation and storage of SOC in arable soil is becoming increasingly important because of the beneficial effects on soil fertility and subsequent improvements in crop yield, not to mention the mitigating effects on climate change (Baveye et al., 2018; UNEP, 2017). In much of China, wheat and maize yield responses reach a plateau at SOC levels of 21.8–46.2 and 22–44.4 Mg ha^−1^, respectively (Wang et al., 2020). Soil and crop management practices have a significant effect on C and N cycles in cultivated land, altering the quantity and quality of crop residues in the soil as well as the overall supply of nutrients (Dou et al., 2018; McDaniel et al., 2014; Tian et al., 2015). In agricultural ecosystems, fertilizer management is an important factor that affects SOC sequestration, crop production and food security (Ren et al., 2021).

The N dynamics affects C sinks through the processes of biological, physical and chemical about fine root turnover, litter decomposition, soil respiration and photosynthetic characteristics (Mandal et al., 2022). Terrestrial C–N interactions are extremely important in determining the long-term sustainability of C sinks in land ecosystems (Deng et al., 2016). The N dynamics are thought to be an important determining factor in terms of long-term C sequestration in agroecosystems, influencing the concentration of plant available nutrients, promoting the crop growth of aboveground and belowground, and affecting the biodegradation of soil organic matter (Li et al., 2015; Meng et al., 2016). In China, a series of long-term experiments was carried out during the 1980s and early 1990s to examine the effects of nutrient management on soil fertility, crop productivity, and nutrient use efficiency (Duan et al., 2014; Yang, Sun & Zhang, 2014). However, few integrated studies have examined the yield and SOC dynamics in deep soil under long-term winter wheat-summer maize cropping systems. Understanding how N enrichment affects cropland C cycling is essential in understanding the effect of increasing N input on global climatic change.

Chemical fertilizer can provide nutrients supply for crop growth, and have becoming a common agricultural management practice for improving the crop yield (Duan et al., 2011; Zhang et al., 2016). In China’s croplands, excessive use of synthetic chemical fertilizers have led to a decrease in soil quality, resulting in a number of adverse agricultural impacts (Liu & Diamond, 2005). Various studies have examined C sequestration and the possibility of mitigating SOC losses by optimizing crop rotation, application rates of inorganic fertilizers and the integration of soil fertility management (Berhane et al., 2020; Srinivasarao et al., 2012). Development of appropriate management practices aimed at sustainable crop productivity and increased SOC sequestration on a regional and global scale is therefore crucial in terms of soil fertility and productivity (FAO, 2011).

Wheat–maize rotation is the principal cropping system in the North China Plain, covering approximately 16 M ha and accounting for about 25% of the total national food production (Yang, Sun & Zhang, 2014). In the past two decades, excessive synthetic chemical fertilizer application during winter wheat-summer maize production, especially in the form of N, has been increased from 7.07 to 26.21 million tons (Guan et al., 2020), influencing the sustainability of future cereal production dramatically (Bei et al., 2018; Chen et al., 2014). Previous studies have examined the changes in SOC levels based on single fertilizer management (Ji et al., 2016), specific crops (Tian et al., 2015), and under single-cropping systems and specific soil types (Zha et al., 2015). However, the long-term effects of different N application levels on SOC and N storage under wheat–maize cropping system need to be quantified in greater detail. The aims of this study, therefore, were to (1) determine the effects of different N application rates on SOC and TN concentrations; (2) compare the C content of wheat and maize biomass during crop rotation; and (3) analyze SOC and N storage and the correlations between SOC and TN with depth.

## Materials and Methods

### Experimental site and climatic conditions

This long-term study was established in July 2012 at Zhangpan Town, Jian’an District, Xuchang City, Henan Province (33°59′N, 113°58′E), China, and field management practices were treated in the same way. Observations for this study were made during the 2017–2018 growing season. The area has a temperate monsoon climate, with annual mean precipitation and temperature of 712 mm and 14.6 °C, respectively. About 60% of the precipitation falls between the months of July and September. The predominant cropping system is double-cropping rotation of winter wheat and summer maize.

The soil type is mortar black soil, with 13.04 and 10.43 g kg^−1^ organic matter, 0.72 and 0.85 g kg^−1^ total N, 0.79 and 0.84 g kg^−1^ total phosphorus, 6.58 and 6.99 g kg^−1^ total potassium, and bulk density were 1.39 and 1.47 g cm^−3^ for wheat and maize in the 0–20 cm soil profile, respectively. Agricultural production levels and the natural conditions of the experimental site are representative of high-yielding winter wheat production in this region.

### Experimental design and field management

The high-yielding wheat and maize cultivars of Aikang 58 and Denghai 678 respectively were used in this study, representing the most commonly planted wheat and maize cultivar in Huanghuai area, wheat and maize were sown in sequence in the same plot. The field experiment consisted of three replicates for each N treatment applied in a randomized block design with four N application rates of 0, 180, 240, and 300 kg ha^−1^ (N0, N180, N240, and N300, respectively). Each plot was 10 × 11 m, with a seeding rate of 187.5 and 30 kg ha^−1^ for wheat and maize, respectively. At the time of sowing, chemical fertilizer comprising 60% urea and 40% sulfur-coated urea was applied by hand before plowing at a rate of 66 kg P ha^−1^ and 72 kg K ha^−1^. Prior to sowing, plowing was carried out to remove pests and weeds, and during the growing period, weeds were hand-hoed several times.

### Soil samples and preparation

After the wheat and maize were harvested, the root system was excavated with shovel to a size of 20 cm (length) × 20 cm (width) × 40 cm (depth) then washed with deionized water, and the above-ground parts were sampled with scissor or axe from the root. Soil samples were collected from the soil layers (every 20 cm up to a depth of 60 cm) in all 12 subplots on May 28 and September 28, 2018 subsequently. Three soil cores (inner diameter: 5.0 cm) were randomly sampled from each subplot then mixed to form one fresh sample for each depth increment. Visible plant material was removed then soil samples were divided into three parts and transported to the laboratory, where they were air dried before passing through a two mm screen. Roots and other debris were then removed before further analysis. The bulk density (BD) of the soil was measured in different layers using a soil bulk sampler with a stainless steel cutting ring (inner diameter: 5.0 cm, height: 5.0 cm) at points adjacent to the soil sampling plots. First, the volume of each soil core was measured then the dry mass was determined after oven drying at 105 °C.

### Analysis of soil physical and chemical properties

The dichromate oxidation method was used to measure the SOC and the Kjeldahl method (Kjeltec 2300 Analyzer Unit; Foss, Hogana, Sweden) was used to analyze the TN (Bremner, 1996; Nelson & Sommers, 1982). Soil pH was measured using a pH meter after shaking for 30 min with a soil: water suspension of 1: 2.5 w/v. Each measurement was performed in triplicate.

### Analysis of soil C and N stocks and soil C and N sequestration

Since no coarse fraction (>2 mm) was found in the soil samples, the following equation was used to calculate the soil C stock (C_s_, Mg ha^−1^):

(1) \begin{eqnarray*}& & {\mathrm{C}}_{\mathrm{s}}=(\mathrm{SOC}\times \mathrm{BD}\times \mathrm{D})/10\end{eqnarray*}

where BD is the soil bulk density (g cm^−3^), SOC is the soil organic carbon concentration (g kg^−1^) and D is the depth of the sampled soil layer (cm). The following equation was used to calculate the soil N stock (N_s_, Mg ha^−1^):

(2) \begin{eqnarray*}& & {\mathrm{N}}_{\mathrm{s}}=(\mathrm{TN}\times \mathrm{BD}\times \mathrm{D})/10\end{eqnarray*}

where BD and D are as above and TN is the total N concentration (g kg^−1^) of the soil.

The C_s_ and N_s_ values were then used to determine C and N sequestration (ΔC_s_ and ΔN_s_, Mg ha^−1^) after maize planting as follows:

(3) \begin{eqnarray*}& & \Delta {\mathrm{C}}_{\mathrm{s}}={\mathrm{C}}_{\mathrm{m}}-{\mathrm{C}}_{\mathrm{w}}\end{eqnarray*}

(4) \begin{eqnarray*}& & \Delta {\mathrm{N}}_{\mathrm{s}}={\mathrm{N}}_{\mathrm{m}}-{\mathrm{N}}_{\mathrm{w}}\end{eqnarray*}

where C_m_ is the soil C stock after maize planting (Mg ha^−1^), C_w_ is the C stock after wheat planting (Mg ha^−1^), N_m_ is the soil N stock after maize planting (Mg ha^−1^) and N_w_ is the N stock at maize planting (Mg ha^−1^).

### Statistical analyses

Data were analyzed using SPSS software version 17.0 (IBM Corp., Armonk, NY, USA). Duncan’s multiple range test was used for variance analysis and to compare mean values (*n* = 3) at a significance level of 0.05.

## Results

### Crop yield

**Figure 1:**
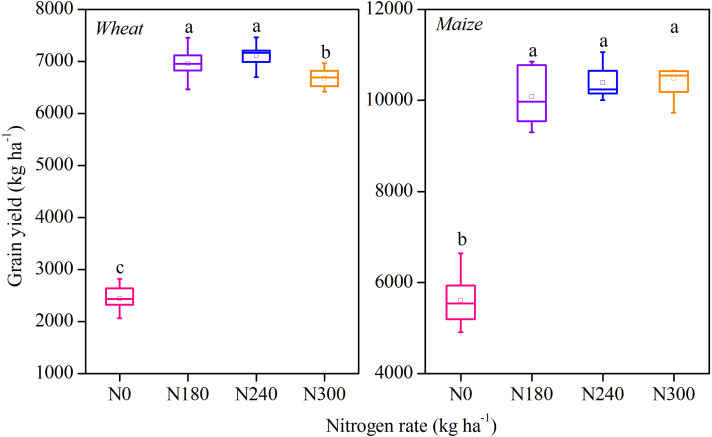
Annual mean grain yield in winter wheat (left panel) and summer maize (right panel) after long-term application of different nitrogen rates (N0, N180, N240 and N300). The box represents ± SE, the whisker represents the Min-Max, the small square represents the mean values, and the horizontal line is the median.

For wheat field, the grain yields under N180, N240 and N300 were significantly higher than that under N0 by 184.67, 191.11 and 173.24%, respectively; the yields under N180 and N240 were significantly higher than that under N300 (*P* < 0.05). For maize field, the grain yields under N180, N240 and N300 were significantly higher than that under N0 by 79.79, 85.23 and 86.85%, respectively (*P* < 0.05); there were no significant differences among N180, N240 and N300 (Fig. 1).

### The SOC and TN content

**Figure 2:**
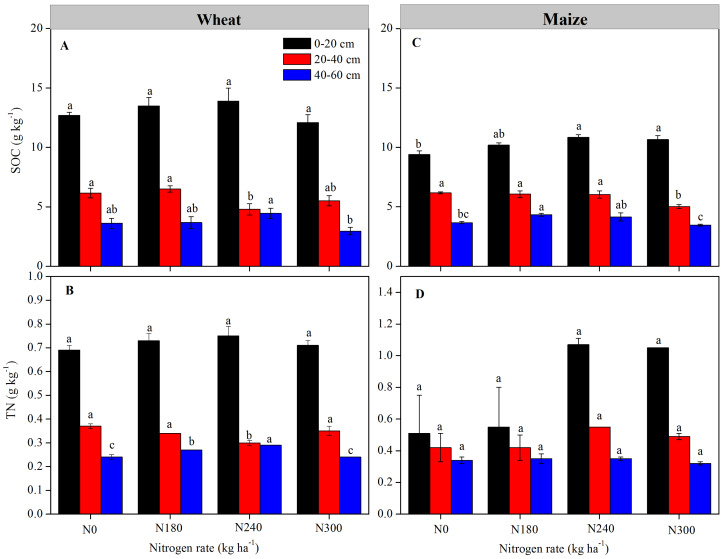
(A & C) Soil organic carbon (SOC) and (B & D) total nitrogen (TN) levels in each soil layer under each nitrogen application rates. Values represent means ± SE. Different lowercase letters above the bars represent significant differences in the same soil layer between different nitrogen levels (*P* < 0.05).

In wheat growing season, SOC and TN were highest in 0–20 cm soil layer, with no significant differences among N levels, the TN was significantly lower under N240 than that under other N levels in 20–40 cm layer, but was significantly higher in 40–60 cm layer (*P* < 0.05) (Fig. 2). In maize growing season, SOC and TN were also highest in 0–20 cm, the SOC under N180, N240 and N300 were significant higher than that under N0 in 0–20 cm layer, the values under N180 and N240 were significant higher than N300 in 20-40 and 40–60 cm (*P* < 0.05). In the entire profile, SOC values were higher after wheat planting than maize planting, while TN were higher under N240 and N300 after maize planting than that after wheat planting.

### The SOC and N storage

**Figure 3:**
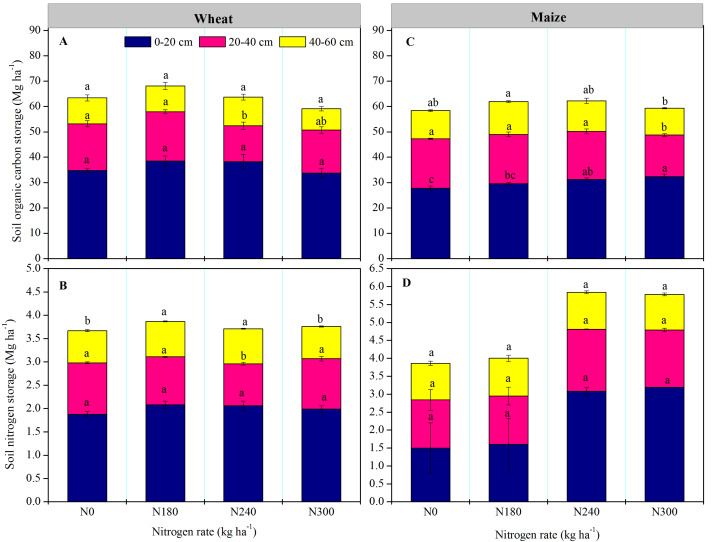
(A & C) Soil organic carbon (SOC) and (B & D) total nitrogen (TN) storage in each soil layer under each nitrogen application rate. Values represent means ± SE. Different lowercase letters above the bars represent significant differences in the same soil layer between different nitrogen levels (*P* < 0.05).

In wheat growing season, SOC and TN storage were highest in the 0–20 cm soil layer, the SOC and TN storage were significant lower than that under other N treatments in 20–40 cm under N240, and the TN storage were significant higher in 40–60 cm under N180 and N240 (*P* < 0.05) (Figs. 3A and 3B). The SOC and TN storage was highest in the entire profile under N180. In maize growing season, the SOC storage was significant higher in 0–20 cm layer under N300, but that under N0, N180 and N240 were significant higher than that under N300 in 20–40 cm (*P* < 0.05) (Fig. 3), the SOC storage was highest in the entire profile under N180, with lowest values under N300. In contrast, TN storage was higher under N240 and N300. The SOC storage in the entire profile was higher after wheat planting than maize planting under all N levels, while TN storage was higher after maize planting, especially under N240 and N300 treatment.

**Figure 4:**
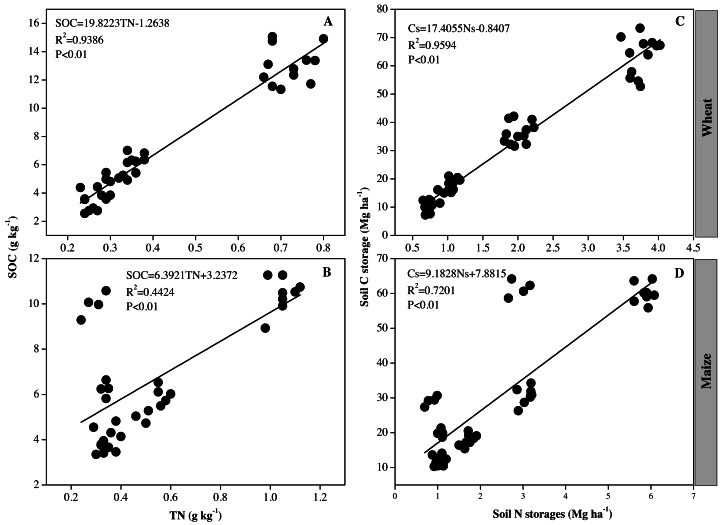
(A–D) Correlations between soil organic carbon (SOC) and total nitrogen (TN) contents, and SOC and TN storage.

### Relationships between SOC and TN content, and SOC and TN storage after wheat and maize planting

The SOC and TN content plus SOC and TN storage were significantly positively correlated, respectively (*P* < 0.01, Fig. 4). Correlations between soil SOC and TN, and soil C and N storage were also stronger after wheat planting than maize planting. Moreover, the correlations between SOC and TN storage were greater than those between SOC and TN content in wheat and maize growing seasons.

### The biomass, SOC content and storage of above and belowground organs

The biomass of wheat and maize were significant increased after adding N, but the SOC content and storage have no significant difference among N treatments for wheat. For maize,the SOC storage of above and belowground were significant higher under N180 and N240 than that under N0 treatment, and the SOC content was highest under N240 than that under other N treatments (Table 1).

### Differences in SOC and TN sequestration after maize planting

**Figure 5:**
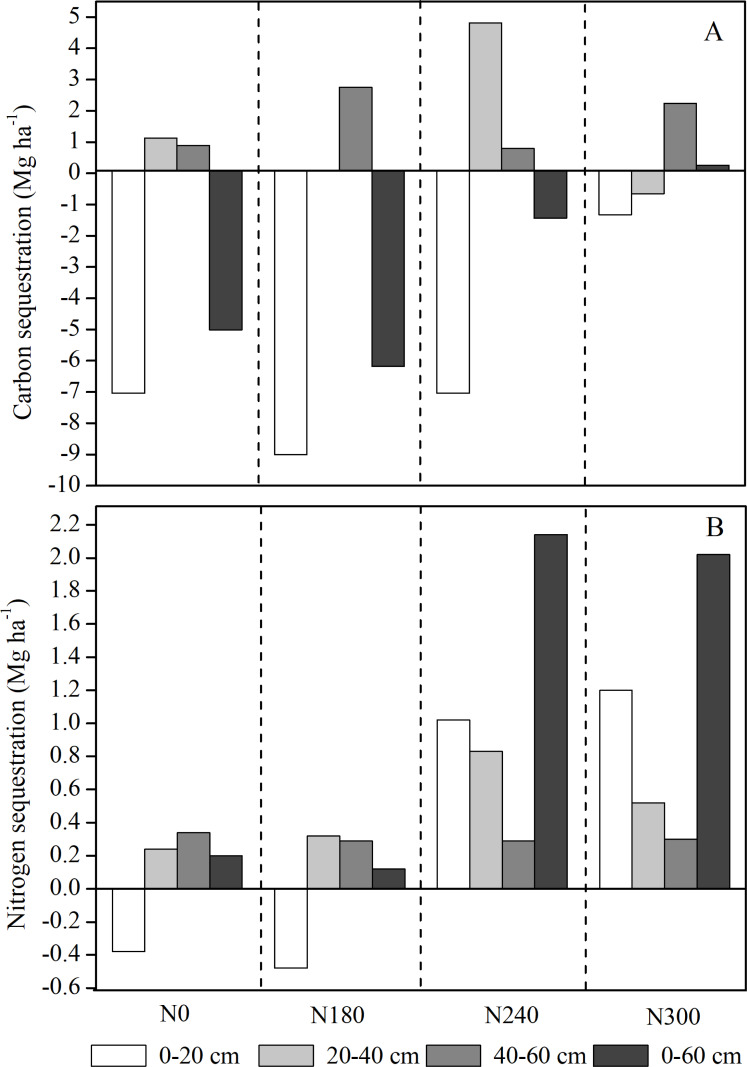
(A) Carbon and (B) nitrogen sequestration in each soil layer under each nitrogen level after maize planting.

The SOC and TN sequestration were different in soil depths and N levels (Fig. 5). The SOC sequestration in the 0–20 cm soil layer decreased following maize harvest , while in the subsoil layers (>20 cm), SOC and TN sequestration increased under almost all N levels. The SOC sequestration was highest in the 20–40 cm layer under N240 (4.81 Mg ha^−1^), followed by the 40–60 cm under N180 treatment. In the 20-40 and 0–60 cm soil layer, TN sequestration was highest at N240 level. The higher TN sequestration were observed under N240 and N300 compared to N0 and N180 across 0-60 soil profile.

### Correlations between SOC, TN storage and soil properties

The SOC (or TN) storage and SOC (or TN) content were significantly positively correlated at each soil depth in both wheat and maize; in wheat, SOC storage and C/N were significantly positively correlated across the entire soil profile. No significant correlations were observed between SOC storage and BD in maize or between N storage and SOC content in both wheat and maize (Table 2).

## Discussion

### Effect of N fertilizer on crop yield

The findings of this study revealed that annual N application increased crop yield compared with control treatment (N0) during the study period. Mean wheat yield was in the order of N240 > N180 > N300 > N0, with significantly higher yield under N180 and N240, similar to a previous report (Wang et al., 2018). In contrast, maize yield was highest under N300 treatment, and was in the order of N300 > N240 > N180 > N0, suggesting that maize has a higher demand for N fertilizer. It was previously revealed that fertilizer treatment significantly increased crop production compared to no fertilizer application (Chen et al., 2015), although the yields under different fertilizer treatments were not significant in both wheat and maize, thus differing from our results. This difference was attributed to the larger amount of crop stubble returned to the soil in the fertilized plots as a result of the increase in crop yield. In other words, a larger grain yield, especially in terms of straw and stalk biomass, inevitably leads to an increase in residue return to the soil compared to no fertilizer or synthetic fertilizer treatment (Huang et al., 2010; Xie et al., 2017), thereby increasing C sequestration.

**Table 1:** The biomass, carbon content and storage of above and belowground organs of wheat and maize under nitrogen treatments.

Component	N0			N180			N240			N300		
	Biomass	Carbon content	Carbon storage	Biomass	Carbon content	Carbon storage	Biomass	Carbon content	Carbon storage	Biomass	Carbon content	Carbon storage
	(Mg ha^−1^)	(g kg^−1^)	(Mg ha^−1^)	(Mg ha^−1^)	(g kg^−1^)	(Mg ha^−1^)	(Mg ha^−1^)	(g kg^−1^)	(Mg ha^−1^)	(Mg ha^−1^)	(g kg^−1^)	(Mg ha^−1^)
Wheat												
Aboveground	2.69^b^	402.33^a^	1.83^a^	7.75^a^	421.93^a^	2.59^a^	7.31^a^	455.5^a^	2.78^a^	7.11^a^	419.2^a^	3.16^a^
Belowground	0.22^b^	427.5^a^	0.32^a^	0.79^a^	378.6^a^	0.21^a^	1.04^a^	337.47^a^	0.21^a^	0.97^a^	382.80^a^	0.39^a^
Total	2.90^b^	–	2.16^a^	8.53^a^	–	2.80^a^	8.34^a^	–	2.99^a^	8.08^a^	–	3.55^a^
Maize												
Aboveground	4.67^b^	359.49^a^	1.68^b^	6.58^a^	397.75^a^	2.63^a^	7.29^a^	388.46^a^	2.84^a^	7.66^a^	426.32^a^	3.26^a^
Belowground	1.17^b^	323.34^b^	0.38^b^	1.90^a^	347.75^ab^	0.66^a^	1.83^a^	378.85^a^	0.69^a^	1.65^a^	263.48^c^	0.43^b^
Total	5.84^b^	–	2.06^b^	8.48^a^	–	3.29^a^	9.12^a^	–	3.53^a^	9.30^a^	–	3.69^a^

**Notes.**

Different lowercase letters represent significant differences among nitrogen treatments (*P* < 0.05). Aboveground parts exclude the panicle grain.

**Table 2:** Pearsons’s correlation coefficients between soil C and N storage and soil properties (C/N: carbon/nitrogen and BD: bulk density) at each soil depth.

Crop		Soil properties							
Wheat	Soil layer	Soil carbon storage				Soil nitrogen storage			
		SOC	TN	C/N	BD	SOC	TN	C/N	BD
	0–20 cm	0.984^**^	0.316	0.730^*^	−0.364	0.402	1.000^**^	−0.105	−0.252
	20–40 cm	0.658^*^	0.699^*^	0.324	0.320	0.082	1.000^**^	−0.321	0.167
	40–60 cm	0.767^**^	0.296	0.794^**^	−0.514	0.463	1.000^**^	0.252	−0.584^*^
	0–60 cm	0.984^**^	0.242	0.901^**^	−0.127	0.216	1.000^**^	−0.186	−0.340
Maize	0–20 cm	0.947^**^	0.391	−0.360	0.163	0.356	0.998^**^	−0.988^**^	0.262
	20–40 cm	0.993^**^	−0.363	0.671^*^	−0.046	−0.354	0.997^**^	−0.944^**^	−0.242
	40–60 cm	0.991^**^	0.184	0.742^**^	−0.157	0.097	0.982^**^	−0.601^*^	−0.092
	0–60 cm	0.957^**^	−0.218	0.336	0.200	−0.213	0.998^**^	−0.992^**^	−0.100

**Notes.**

* Significant correlations at *P* = 0.05 (*P* < 0.05) (2-tailed) in a sample size of *n* = 12.

** Significant correlations at *P* < 0.01 (2-tailed) in a sample size of *n* = 12.

### Content and storage of SOC and N

Soil organic C affects soil functions and processes, thereby affecting nutrient availability for plants (Troeh & Thompson, 2005). Fertilization effects on SOC show regional differences, and in our study, SOC and TN content decreased gradually with increasing soil depth under all N treatments, with highest contents in the 0–20 cm layer. This is thought to be because the rate of decay of soil C is faster in deeper soil layers, consistent with previous findings in northeast China and the Loess Plateau (Wang et al., 2011a; Wang, Wang & Zu, 2014). In northern China, soil organic C and N conditions in both upper and deeper soil layers are significantly affected by N levels, and in this study, TN values were higher under N240 and N300 treatment during the maize compared to wheat season. This is probably why maize yield increased with increasing N fertilizer application. Moreover, SOC values were higher during the wheat compared to maize season in the entire profile. This is thought to be because increased rainfall during the maize season have resulted in a relatively higher soil moisture content, resulting in a more favorable soil environment for rapid propagation of soil microorganisms, and subsequent acceleration of SOC mineralization and decomposition (Silver & Miya, 2001; Wang et al., 2020; Zhang et al., 2010). In contrast, during the winter wheat growth period, rainfall was lower, and activity of microorganisms in the soil was likely to be lower, thereby slowing the rate of SOC decomposition. Moreover, wheat stubble returned to the maize field has a higher ratio of C: N than maize stubble, which is also likely to have had an effect on SOC decomposition (Zhao et al., 2016).

Cropping systems have a significant effect on SOC stocks, influencing the balance between C input from litter and C losses *via* decomposition (Huang, Sun & Zhang, 2012). In general, soil depth is stable, suggesting that soil C stocks are determined by SOC and soil BD (Deng et al., 2013). In this study, soil SOC and TN storage was highest in the 0–20 cm layer under all N levels in both wheat and maize. Nutrient and organic matter accumulation in the topsoil is the result of complex interactions between biotic processes regulated by plants and soil biota, and abiotic processes driven by environmental processes (Hooper, Bignell & Brown, 2000). Land use and the depth of sampling are therefore important in terms of SOC and soil C measurements (Strahm et al., 2009; VandenBygaart et al., 2010). In this study, SOC and TN storage were high under N180 for wheat and SOC storage showed no further increases with increasing N application. Soil nutrients are gradually depleted during continuous cropping under no fertilizer application, thereby affecting SOC dynamics and stability (Zhang et al., 2010). Thus, overall, balanced chemical fertilizer use combined with straw application is the most beneficial to SOC stocks.

### Correlations between SOC and TN content, SOC and TN storage after wheat and maize planting

After wheat and maize planting, soil SOC and TN, and soil C and N storage were significantly positive correlated (*P* < 0.01). Deng et al. (2018) also observed a significant positive correlation between soil C and N on the Loess Plateau in vegetation succession, while a significant positive effect of N enrichment on SOC was also revealed in globally syntheses (Liu & Greaver, 2010; Lu et al., 2011). The correlations of soil C and N storage were different between wheat and maize planting, probably because SOC stocks are affected by a number of soil chemical and physical properties (Wang et al., 2013), and they differed following long-term N treatments in this study.

### The biomass, SOC content and storage of aboveground and belowground organs

Different crop types result in different litter decomposition processes, thereby affecting the release of C and N into the soil (Zhang et al., 2013). Aboveground vegetation plays an essential role in regulating the biogeochemistry of different ecosystems, fixing C and nutrients and preventing nutrient losses following disturbance. In this study, the above and belowground biomass of both wheat and maize were significantly higher following N application compared to N0 treatment. It is well known that N fertilization has a positive effect on plant growth, with aboveground net primary productivity and belowground biomass both increasing in response to increasing N application (Peng, Guo & Yang, 2017; Tang et al., 2017).

In this study, the SOC content and storage of above and belowground wheat organs did not significantly differ between N treatments, similar to a previous report showing that the level of N application had no effect on SOC, with N addition increasing aboveground plant productivity but not root input into the soil C pool (King et al., 2005). Meanwhile, in maize, no significant differences in aboveground SOC was observed between N treatments, although belowground SOC content and storage was significantly higher under N180 and N240. A significant increase in C stocks with N application was previously observed in both above and belowground biomass, suggesting that N treatment stimulates biomass accumulation, thereby increasing C and nutrient retention in the soil (Lu et al., 2011). Overall, these findings suggest that results differ between crop types.

### Differences between SOC and TN sequestration under different N levels after maize planting

The N levels and soil layers had a significant effect on SOC and N sequestration. The primary productivity of litter and roots have a significant effect on soil carbon sequestration, resulting primarily in an accumulation of SOC and N storage in the topsoil layer (Cheng et al., 2006; Dou et al., 2018). In this study, soil SOC sequestration in the 0–20 cm soil layer decreased under all N treatments, while both SOC and TN sequestration almost tended to increase in the 20–60 cm layer after maize planting for all N treatments. This is thought to be because larger maize roots are distributed at a depth of 20–30 cm (Dou et al., 2016), while soil C and N in the topsoil layer are lost rapidly, possibly due to soil respiration and incomplete decomposition soil organic matter in residues (Cheng et al., 2006). The N dynamics are another key factor affecting long-term terrestrial C sequestration (Luo, Field & Jackson, 2006). In this study, SOC and TN sequestration were at their highest in the 20–40 cm layer at N240 under all the treatments. The SOC sequestration almost tended to decrease in the 0–60 cm soil profile under N treatments; however, N sequestration were all increased, and was significantly higher under N240 and N300 treatment. These findings suggest that the consumption of SOC is greater than production, with combined application of N fertilizer and straw working together to increase N sequestration during the maize growing season. In addition, soil particle size, and the composition and dynamics of microbial communities as well as the increase in biomass with increasing N levels also have an effect on the biogeochemical cycles of soil carbon and N (Cleveland et al., 2004; Li et al., 2005), thereby increasing rates of carbon sequestration.

### Pearson’s correlation coefficients between SOC, TN storage and soil properties

**Figure 6:**
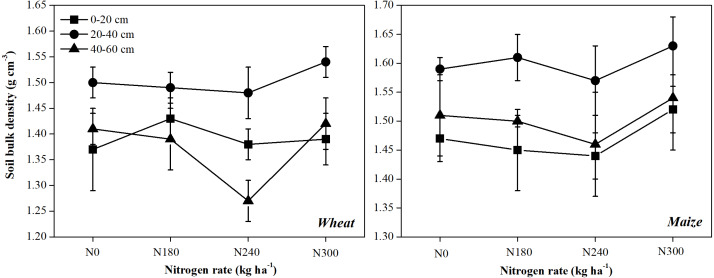
Dynamics of soil bulk density in at each soil depth under each nitrogen application rate.

In this study, SOC and TN storage and contents of SOC and TN were significantly positively correlated in each soil layer after both wheat and maize planting, suggesting that SOC and TN storage are the result of soil C and N accumulation. Meanwhile, there were respective negative correlations between SOC and TN storage and BD, except in the 20–40 cm layer after wheat planting and the 0–20 cm layer after maize planting (Table 2). Wang et al. (2011b) revealed a negative relationship between SOC and TN with soil bulk density, while Singh et al. (2012) and Singh, Singh & Singh (2012) reported a significant increase in SOC and TN but a significant decrease in BD during the restoration of degraded land. In this study, BD differed between soil layers (Fig. 6), suggesting that the differences between studies were due to differences in soil characteristics and/or the crop type.

## Conclusions

This study investigated the SOC and TN characteristics of wheat-maize cropping system under different N application rates. The findings of this study suggest that long-term application of chemical fertilizers could significantly improve yield, especially after N180 and N240 treatments. The SOC and TN decreased gradually with increasing soil depth under four N treatments, SOC values were higher after wheat than that after maize planting. Correlations between SOC and TN content, SOC and N storage were stronger after wheat than that after maize planting. Meanwhile, SOC and TN sequestration were lower under N180 than that under other N treatments after maize planting, suitable N application aiding improvements in crop quality and efficiency.

## Supplemental Information

10.7717/peerj.13568/supp-1 Supplemental Information 1 Raw data for Figures 1–6

## Additional Information and Declarations

### Data Availability

The following information was supplied regarding data availability: The raw measurements are available in the Supplementary File.
